# Combined Effects of Isokinetic Training and Botulinum Toxin Type A on Spastic Equinus Foot in Patients with Chronic Stroke: A Pilot, Single-blind, Randomized Controlled Trial

**DOI:** 10.3390/toxins11040210

**Published:** 2019-04-08

**Authors:** Nicoletta Cinone, Sara Letizia, Luigi Santoro, Salvatore Facciorusso, Raffaella Armiento, Alessandro Picelli, Maurizio Ranieri, Andrea Santamato

**Affiliations:** 1 Spasticity and Movement Disorders “ReSTaRt” Unit, Physical Medicine and Rehabilitation Section, OORR Hospital, University of Foggia, 71122 Foggia, Italy; n.cinone@gmail.com (N.C.); sara.letizia@live.it (S.L.); luigisantoro@alice.it (L.S.); s.facciorusso89@gmail.com (S.F.); armientoraffaella@alice.it (R.A.); 2 Neuromotor and Cognitive Rehabilitation Research Center, Department of Neurosciences, Biomedicine and Movement Sciences, University of Verona, 37134 Verona, Italy; alessandro.picelli@univr.it; 3 Physical Medicine and Rehabilitation Section, “OORR Hospital”, 71122 Foggia, Italy; maurizio.ranieri@unifg.it

**Keywords:** botulinum toxin A, isokinetic, poststroke, spasticity, BoNT-A, equinus foot

## Abstract

Botulinum toxin A (BoNT-A) has been shown effective for poststroke lower limb spasticity. Following injections, a wide range of multidisciplinary approach has been previously provided. The purpose of this pilot, single-blind, randomized controlled trial was to determine whether BoNT-A combined with a regime of a four-week ankle isokinetic treatment has a positive effect on function and spasticity, compared with BoNT-A alone. Secondly, the validity of the use of an isokinetic dynamometer to measure the stretch reflex at the ankle joint and residual strength has been investigated. Twenty-five chronic stroke patients were randomized to receive combined treatment (*n* = 12; experimental group) or BoNT-A alone (*n* = 13; control group). Outcome measures were based on the International Classification of Functioning, Disability and Health. An isokinetic dynamometer was also used for stretch reflex and strength assessment. Patients were evaluated at baseline (t0), after five (t1) and eight weeks after the injection (t2). The experimental group reported significantly greater improvements on lower limb spasticity, especially after eight weeks from baseline. Gait speed (10-m walk test) and walking capacity (6-min walking test) revealed statistically significantly better improvement in the experimental than in control group. Peak resistive ankle torque during growing angular velocities showed a significant reduction at the higher velocities after BoNT-A injections in the experimental group. Peak dorsiflexor torque was significantly increased in the experimental group and peak plantarflexor torque was significantly decreased in control group. Alternative rehabilitation strategies that combine BoNT-A and an intense ankle isokinetic treatment are effective in reducing tone and improving residual strength and motor function in patients with chronic hemiparesis.

## 1. Introduction

Stroke is one of the leading causes of mortality and a major cause of disability worldwide. Spasticity, with an occurrence after stroke of between 18% and 38%, may interfere with the execution of daily activities, social participation, and quality of life [1].

In patients with lower limb spasticity after stroke, spastic equinus foot represents a prolonged abnormal lower limb posture and affects gait, standing, and transfer [2]. This deformity is reported in 18% of stroke patients [3]; spasticity of posterior muscles of the leg and weakness (especially the tibialis anterior and the peroneus muscle) with tendon shortening complicate balance and gait.

Calf muscle spasticity typically causes foot deformity, which results in the loss of heel strike, reduced toe clearance, and an inadequate base of support. These impairments decrease multiple aspects of gait ability: cadence, stride length, speed, capacity, and stability [4,5].

In the management of poststroke lower limb spasticity, the efficacy and safety of botulinum toxin A (BoNT-A) injections were investigated by many randomized controlled studies [6,7,8,9,10,11]. Botulinum neurotoxins (BoNTs) are produced by sporulating and anaerobic Gram-positive bacteria of the genus Clostridium, which consists of more than 150 species. Using antisera from animals immunized with specific toxin types, BoNTs have been classified into seven different serotypes indicated with alphabetical letters (from A to G), but two toxin types—type A and type B—are used in the clinical setting [12]. BoNT-A reduces spasticity in selected muscles by blocking acetylcholine release at the neuromuscular junction [13,14]. BoNT-A injection into plantar flexor muscles reduces muscle tone and improves ankle range of motion. Many muscles are responsible for equinus foot deformity (e.g., medial and lateral gastrocnemius, soleus, tibialis posterior, flexor hallucis longus and brevis, flexor digitorum longus and brevis, and extensor hallucis brevis), but since the gastrocnemius and the soleus are mainly involved in this posture, BoNT-A treatment targets these muscles to reduce the drive to plantarflexion [15,16]. However, it is still unclear whether BoNT-A can improve voluntary control of residual strength or gait ability in chronic stroke patients [17,18,19]. A systematic review of gait velocity in RCTs reported a 0.044 m/s increase (an effect size of 0.193) in gait velocity in the treatment groups, although the number of studies reporting such an improvement was small [20]. It is generally assumed that negative signs of upper motor neurone syndrome are associated with more disability and represent a big challenge for rehabilitation [21,22]. Multidisciplinary approach in the management of poststroke spasticity has been considered and several trials supporting the role of adjunctive therapies after BoNT-A injections, have been published [23,24,25,26,27].

Hence, even if the association of BoNT-A with concomitant treatment is widely accepted, there is no general agreement on which adjunctive treatment is more effective in terms of functional walking [28]. Among the wide range of possibilities, there is only a paucity of published studies, often dated, concerning the role of isokinetic treatment for gait function in poststroke patients. To our knowledge, no study has combined BoNT-A treatment with an intensive isokinetic training of the ankle for spastic equine foot [29,30]. From our point of view, strengthening paretic foot in patients with stroke could be a key issue for improving gait and functional parameters.

Aim of the study is to evaluate the effectiveness of a combined BoNT-A treatment and ankle isokinetic training in subjects with spastic equinus foot after stroke. Conscious of the CNS neuroplastic behavior after cerebrovascular accident, it has been hypothesized that repeated ankle dorsiflexion, combined with a coincident voluntary effort, could help to restore the synaptic spinal connections, thus improve the motor function of the lower limbs, the spatiotemporal parameters of gait, as well as the activity and participation of subjects with chronic stroke-related motor impairment. 

Secondary objective is also to verify the applicability of the isokinetic dynamometer both for measuring spasticity (peak resistive torque) and residual strength (concentric peak torque). Based on previous studies, we hypnotized that BoNT-A injections would reduce stretch reflex intensity, decrease plantiflexor muscle strength, and increase ankle dorsiflexor muscle strength [29,31]. 

## 2. Results

A total of 25 outpatient subjects were included in this pilot, single-blind, randomized controlled study. Table 1 presents demographic characteristics at the time of enrolment.

No significant differences were found between experimental and control group for age, sex, time since stroke, side of hemiplegia, and mean dose of BoNT-A injected per muscle (Table 2, *p* > 0.05).

Table 3 summarizes the results related to body structure. The comparison between MAS scores, reported as medians, shows a significant reduction in both groups at t1 and t2 compared to baseline.

MI did not differ significantly between assessment periods. A significant decrease in the mean TSA therefore indicates a reduction in spasticity in experimental and control group after five weeks, that persists in between groups analysis t0–t2 for soleus muscle (*p* = 0.042). 

In terms of functional and spatiotemporal parameters, there also was a significant change in 10mWt at t0–t1 (*p* 0.018, z −2.37) and t0–t2 (*p* 0.027, z −2.21) in the experimental group, but not in control (Table 4). The 6MWT showed a statistically significant change at t0–t1(*p* 0.022, z −2.37) and t0–t2 (*p* 0.034, z −2.21) just in the experimental group. The cadence, step length, and duration of single-phase support were better than baseline in both groups, but significance was found in the experimental group after five weeks for cadence (*p* 0.018) and duration of single-phase support in nonaffected side (*p* 0.04).

Peak resistive torque (rPT) during growing angular velocities showed a significant reduction at the higher velocities (Table 5) after BoNT-A injections (experimental 90°/s: *p* = 0.025, control *p* = 0.046; experimental 180°/s *p* = 0.042, control *p* = 0.047). Furthermore, significant improvement of rPT was achieved in isokinetic training group at t0–t2 both for 90°/s (*p* = 0.045) and 180°/s (*p* = 0.047). Peak concentric dorsiflexion torque was significantly increased at 60°/s in experimental group at t0–t1 (*p* = 0.002) and t0–t2 (*p* = 0.034). Peak plantarflexion torque was significantly decreased at 60°/s in experimental group at t0–t1 (*p* = 0.005). Peak concentric dorsiflexion torque was significantly increased at 60°/s in control group at t0–t1 (*p* = 0.042) and t0–t2 (*p* = 0.034). Peak plantarflexion torque was significantly decreased at 60°/s in control group both at t0–t1 (*p* = 0.042) and t0–t2 (*p* = 0.032).

## 3. Discussion

BoNT-A is a well-established treatment in the management of poststroke spasticity, but evidence, in terms of functional gain during gait, is still debated, considering that only few studies showing effective improvements and others reporting not relevant results on its functional efficacy [19,32,33]. With both muscular weakness and spasticity considered to be significantly detrimental to functioning in poststroke patients, there is a call for research to determine the efficacy of treatment for these motor impairments. This study is the first randomized controlled study to evaluate the combined effects of isokinetic training and botulinum toxin injection in the treatment of chronic spastic foot after stroke. Our results showed that patients allocated to the experimental group (BoNT-A + isokinetic training) obtained significantly greater reduction in muscle overactivity as measured by rPT and TSA than control group. A previous recent study showed similar results, confirming that BoNT-A clearly reduces knee extensor stretch reflex intensity as demonstrated by the decrease in peak resistive torque for all three velocities tested [29].

Moreover, interestingly, the results of this study confirmed our initial hypothesis: that prolonged passive and active training of agonist muscles, obtained by isokinetic dynamometer, gives a potential benefit and improves functional gait performance. The patterns of dysfunction most commonly treated with BoNT-A in the affected lower limb are the equinovarus and equinus foot. In particular, the equinus foot is the most common spastic deformity causing gait impairment in patients with upper motor neuron syndromes [34,35,36]. In both the experimental group and in the control group there is an evident reduction in the tone in terms of MAS, and especially of TSA. From the elaboration of the data related to the stretch reflex, it is intuitive as increasing the angular velocity a greater muscular response to the stretch is induced, since spasticity is, by definition, velocity-dependent. Following neuromuscular blockade with BoNT-A, the joint gain detected by clinical assessment becomes a value that significantly decreases five weeks after the injection. The rPT values of the plantar flexor muscles measured at eight weeks from baseline retain a significant difference only in the experimental group. The persistence of a lower resistance offered to the fast stretching could be related to the inclusion in the protocol, of passive ankle mobilizations with lower angular velocities. In a 2016 Hungarian study, the authors demonstrated that fifteen minutes of passive ankle mobilization in stroke patients with a mechanical device, are able to induce an increased BOLD (blood oxygen level-dependent) in fMRI not only in the ipsilateral area of the lesion (S1, M1, and SMA), but also in the supramarginal gyrus and the contralateral S2 area [37]. If the reduction of spasticity is widely known, the existing literature on gait function is controversial. Spastic hypertonia of the plantar flexor muscles can lead to equinus foot deformity and it is one of the most important factors in walking impairments in stroke patients. Equinus foot deformity also affects the execution of common and essential motor gestures during daily activities, including the sit-to-stand movement, which requires a dorsiflexor engagement greater than just walking or climbing stairs [38].

Previous studies showed that an effective treatment of lower-limb spasticity is important in improving gait ability and enhancing the independence of patients after a stroke [39,40].

A 2009 Canadian study assessed the impact of BoNT-A injections in triceps surae on kinetic and kinematic parameters of the ankle, demonstrating better plantar support in-stance and increased dorsiflexion in mid-stance until ten weeks from treatment [41]. A recent study demonstrated that BoNT-A treatment for lower limb spasticity (medial and lateral gastrocnemius and soleus) combined with intensive rehabilitation, was effective in improving spasticity and the 6MD (6-Minute Walking Distance Test) compared with intensive rehabilitation alone in patients with chronic stroke [30]. A systematic review has recently been published to evaluate the effectiveness of BoNT-A injection on walking and quality of life in poststroke lower limb spasticity. The authors did not find any evidence that supports or refutes improvement on walking or quality of life, based on the review of 107 previous published papers [18]. Sometimes, higher doses are necessary especially in the more severe forms of spasticity in the lower limbs, so that supplementary rehabilitative treatments such as casting, splinting, and taping which implement the antispastic effect are required [25,31]. In our study, we wanted to restore the synaptic connections through the possible retrograde effect of BoNT-A and, once the ankle was released, use the residual strength to obtain an efficient dorsiflexion by isokinetic training. One of the first studies was conducted on twenty patients suffering from poststroke spastic hemiparesis: the authors found an increase in strength of the dorsiflexor muscles equal to 47% and 58% for the plantiflexor muscles after 6 weeks isokinetic strength training [28]. In a nonrandomized, self-controlled trial, authors observed gains in strength (between 15.8% and 153.9%) and gait velocity after 6 weeks isokinetic knee training in chronic stroke patients [42]. Similar results found that bilateral knee–ankle isokinetic strengthening training, in addition to conventional rehabilitation program after stroke, was effective on strengthening muscles on both sides, improving functional parameters, gait, balance, and life quality (Functional Independence Measure, Stroke Specific Quality of Life Scale, Timed 10-Meter Walk Test, Six-Minute Walk Test, Stair- Climbing Test, Timed up&go Test, and Berg Balance Scale) [43].

A study conducted in Taiwan in 2015 compared the effect of isokinetic and isotonic training in two groups of poststroke subjects. The authors found a significant improvement of functional capacity parameters (increase of the flexor muscles’ PT, knee extensors, and peak torque) and quality of life (Short Form-36) in isokinetic training compared to isotonic training [44].

In our study, we observed that patients allocated in the experimental group gained a significantly greater improvement in walking ability as demonstrated by 6MWT and 10mWt.

Moreover, functional results were shown to maintain significance at eight weeks from the first assessment period; in our view, this was probably because patients who performed isokinetic training were more likely to adopt a new functional motor skill and to maintain it than those who did not perform any rehabilitative approach to gait recovery after BoNT-A injection. Isokinetic training after injection in our study focused on voluntary control in the ankle, as known weakness often coexists with spasticity. The results of the study are in line with our hypothesis, the peak resistive torque during passive stretching was reduced, and the angle at which peak resistive torque occurred was increased after BoNT-A injection. A further reflection concerns the paralyzing effect of botulinum toxin in the injected muscles, as demonstrated by the decreased plantarflexion at t1 and t2 in both groups. The muscle is theoretically weakened by blocking nerve impulses to the muscle fibers, and muscle weakness has been reported in experiments with rabbits and cats [45,46]. Few studies have been published in this field. A double-blind, placebo-controlled study reported a decrease of 40% of maximum voluntary grip strength using hand dynamometry after BoNT-A injection in the flexor digitorum muscle [47]. In our study, voluntary control of plantarflexion was not as significant as that found by the authors, most likely because in chronic patients the motor function is already altered by intrinsic muscular modification and rheological properties. The reduction of plantarflexion alone, as pointed out by plantiflexor peak torque, is detrimental to the ability to push off during gait, but the functional parameters during gait were improved, especially in the experimental group. Indeed, our results confirm that combining an intensive ankle rehabilitation program with BoNT-A injection could potentiate dorsiflexor muscle strength and facilitate gait pattern also in chronic patients.

## 4. Conclusions

In light of our results, we can conclude that isokinetic treatment combined with BoNT-A injection in plantarflexor muscles is effective on functional gait parameters and dorsiflexor strength in chronic hemiparetic subjects. Adequate muscle strength is important for maintaining the ability to walk. The main limitation of this study is the absence of a functional assessment, such as a three-dimensional gait analysis or specific outcome measures aimed at evaluating gait ability. Future studies, with more patients, comparing different isokinetic training program after BoNT-A injections, are needed to further validate the present findings.

## 5. Materials and Methods 

Subjects with chronic stroke and spastic hemiparesis admitted to the Department of Rehabilitation Medicine of Foggia University Hospital “OORR” from January 2018 to October 2018, were enrolled according to the inclusion criteria: diagnosis of stroke confirmed by brain computed tomography or magnetic resonance imaging; ≥6 months after stroke onset; age ≥ 20 years; ankle plantar flexor spasticity ≥ 2 on the Modified Ashworth Scale (MAS); insufficient control of ankle dorsiflexion ≤ 3 on the Medical Research Council Scale (MRC); and ≥6-min independent or supervised gait ability with or without an assistive device such as an orthosis or a cane. Exclusion criteria: a previous history of local surgery; BoNT-A injection in the past 4 months; severe ankle contracture or lower limb tendon retraction severe hemodynamic instability; and cognitive disorders or other comorbidities that would affect gait disturbance, and thus interfere with the study.

The patients were randomized in two groups: the experimental group, who received combined BoNT-A injections and isokinetic training, or the control group, who underwent BoNT-A injection alone. Randomizing software was used to allocate patients in both groups. This study was approved by Ethics Committee of Hospital OORR, Foggia, and informed consent was obtained from all subjects.

### 5.1. Outcome Measures

After the first baseline clinical instrumental assessment (t0) and subsequent BoNT-A treatment, patients in the experimental group were initiated for isokinetic training for four weeks, five days a week. Patients were then re-evaluated after five weeks (t1) and eight weeks after the injection (t2).

Patients were examined by the same investigator who was blind to the treatment. The clinical evaluation was based on the International Classification of Functioning, Disability and Health (ICF) and, for the body function and structure ICF domain, the Modified Ashworth Scale (MAS), Motricity Index (MI), and Modified Tardieu Scale (MTS) were used.

MAS was used to assess ankle plantar flexor muscles spasticity and MI to measure strength in lower extremities [48].

Tardieu spasticity angle was measured during the Modified Tardieu Scale, and reflects the velocity-dependent stretch reflex. It is the difference between the angle of arrest at slow speed and the angle of catch-and-release/clonus at fast speed during a passive ankle dorsiflexion [49,50].

The functional tests (activity ICF domain) carried out were the six-minute walk test (6MWT), as a submaximal test of aerobic capacity/endurance, and the 10-Meter Walking test (10mWt), to evaluate walking speed over a short distance [51,52].

Spatial temporal parameters (cadence, step length, and duration of the single support phase in both sides) were recorded through baropodometric measurements and mediated from two trials. Subjects were asked to walk alone on the footboard at their self-selected walking speed in order to recorder spatiotemporal parameters. A Humac NORM cybex (CSMi, Stoughton, MA, USA) isokinetic dynamometer was used for isokinetic training and assessment. Peak resistive torque was recorded during one set of five continuous passive ankle dorsiflexion movement at 10, 30, 90, and 180°/s within 40-degree joint excursion (−10° to 30°). Every set was followed by 30 s rest. Patients were asked to relax and avoid muscle activation on both sides. For the assessment of plantarflexor and dorsiflexor strength, an isokinetic test was performed at an angular rate of 60°/s in the active range of motion evaluated in the paretic side. This velocity was selected because achieved by all patients and in accordance with previous literature [35]. Maximal voluntary strength was assessed during five maximal concentric contractions. It was therefore measured the peak torque (PT) in the two ankle movements.

### 5.2. Interventions

All participants were injected with Onabotulinumtoxin A (BOTOX®, Allergan Inc., Irvine, CA, USA) into the lateral and medial head of the gastrocnemius and in the soleus of the affected lower limb, based on clinical evaluation. BoNT-A dose was adjusted in a range between 50 and 100 U (Royal College of Physician, 2018) for each muscle according to patient’s muscle size, weakness, and response to previous treatment, if performed [53]. Injection was performed under US guidance at two sites per muscle, close to the motor point (MyLab 70 XV, Esaote, Genova, Italy) and linear transducer (scanning frequency, 6–18 MHz).

In addition, patients allocated in the experimental group underwent five 50-min sessions per week for four consecutive weeks, for a total of 20 sessions. Every ankle dorsiflexion program session included 30 continuous passive repetitions at 45°/s and 60°/s, 20-s rest period; 10 repetitions 30°/s, 30-s rest period; 15 repetitions 45°/s, 30-s rest repetitions; 15 repetitions 60°/s, 30-s rest repetitions; 10 repetitions 90°/s; 15 repetitions 45°/s; 30-s rest repetitions. 

The training sessions ended with a 5-min cool-down consisting of mild stretching exercises (−20° to 30°).

### 5.3. Statistical Analysis

The statistical analysis was performed using the Statistics toolbox SPSS 16.00 (SPSS Inc., Chicago, IL, USA) Shapiro–Wilk test was used for determining whether the continuous variables were normally distributed. The comparison of the means and medians of the groups were determined by using Student’s *t*-test and Mann–Whitney U test (for continuous variables). Nonparametric paired Wilcoxon test was carried out to assess within-groups different before and after the treatment in the groups. A *p*-value of <0.05 was considered as statistically significant. 

## Figures and Tables

**Table 1:** Demographic characteristics of enrolled subjects.

Variables	Experimental Group (*n* = 12)	Control Group (*n* = 13)
Age years, mean	56.20 (8.92)	56.40 (7.01)
Sex, n. M/F	8/4	7/6
Side of hemiparesis, n R/L	5/7	5/8
Etiology, Ischaemic/Haemorragic	9/3	8/5
Time since stroke, years, mean	66.42 (37.83)	43.86 (29.84)

Data are reported as mean (SD).

**Table 2:** Mean average injected dosage of Onabotulinumtoxin A.

Injection site	Experimental Group (U)	Control Group (U)
Total dose, Triceps Surae	107.14 (19.70)	120.70 (18.62)
Medial Gastrocnemius	57.14 (18.29)	60.28 (16.50)
Lateral Gastrocnemius	53.33 (14.71)	52.24 (10.04)
Soleus	47.5 (14.33)	53.50 (14.31)

Data are reported as mean (SD) for every single muscle.

**Table 3:** Clinical scales for body structure International Classification of Functioning, Disability and Health (ICF) domain.

Body Structure	t0		t1		t2	
Outcome	Experimental	Control	Experimental	Control	Experimental	Control
MAS GM	2	2	1+	1+	1+	1+
MAS SOL	2	2	1+	1+	1+	1+
MI	51.67 (10.12)	49.18 (10.25)	54.17 (8.65)	52.18 (10.25)	54.17 (9.65)	53.18 (12.09)
TSA GM	19.45 (5.8)	20 (4.6)	13.45 (4.8) *	14.25 (6.6) *	15.78 (5.8)	16.45 (7.2)
TSASOL	13.05 (6.6)	15 (5.9)	9.45 (5.8) *	10.76 (5.4) *	9.03 (5.8) *	11.91 (4.56)

MAS: Modified Ashworth Scale (data are reported as median); GM: Gastrocnemius; SOL: Soleus; MI: Motricity Index; TSA: Tardieu Score Angle. Data are reported as median/mean (SD) *: *p* < 0.05.

**Table 4:** The changes in spatiotemporal parameters in the experimental and control group over 8 weeks.

Activity	t0		t1		t2	
Outcome	Experimental	Control	Experimental	Control	Experimental	Control
10mWt	0.62 (0.22)	0.61 (0.18)	0.69 (0.26) *	0.64 (0.21)	0.65 (0.24) *	0.62 (0.12)
6MWT	221.33 (89.56)	217.23(87.56)	54.17 (8.65)	236 (92.43) *	236 (86.53) *	220.63 (90.46)
Cadence	76.17 (16.10)	76.24 (14.6)	80.37 (13.4) *	78.9 (12.6)	78.41 (13.96)	77.2 (14.26)
Step NP	31.68 (8.5)	32.5 (9.01)	31.10 (8.95)	33.28 (10.12)	32.87 (9.1)	31.54 (10.12)
Step P	30.17 (9.14)	31.75 (8.79)	30.12 (8.96)	31.12 (9.27)	33.69 (8.75)	31.82 (9.75)
SSP NP	1.9 (0.57)	1.92 (0.32)	1.75 (0.45) *	1.89 (0.57)	1.80 (0.58)	1.87 (1.01)
SSP P	1.55 (0.8)	1.51 (0.32)	1.60 (0.27)	1.53 (0.38)	1.58 (0.57)	1.57 (0.37)

10mWt: Ten-meter walk test (m/s); 6MWT: Six-minute walking test (m). Cadence: steps/min; Step: step length (cm); NP: non-paretic side; P: paretic side; SSP: Single support phase. Data are reported as mean (SD). *: *p* < 0.05.

**Table 5:** Stretch reflex and strength assessment in experimental and control group.

Isokinetic	t0		t1		t2	
Outcome	Experimental	Control	Experimental	Control	Experimental	Control
rPT 10°/s	2.35 (0.65)	2.41 (0.62)	2.31 (0.78)	2.35 (0.54)	2.32 (0.76)	2.36 (0.79)
rPT 30°/s	3.25 (1.14)	3.12 (1.12)	3.24 (0.5)	3.09 (0.95)	3.28 (0.81)	3.10 (0.12)
rPT 90°/s	9.1 (1.13)	9.35 (1.25)	8.31 (1.2) *	8.75 (0.97) *	8.5 (1.17) *	9.2 (1.25)
rPT 180°/s	11.7 (1.88)	12.1 (1.79)	10.9 (1.59) *	11.40 (1.35) *	10.91 (1.12) *	11.7 (1.12)
PT df	8.6 (0.15)	8.91 (0.82)	10.82 (1.59) *	9.71 (0.92) *	9.93 (1.45) *	9.28 (0.95)
PT pf	10.09 (0.63)	10.09 (0.63)	9.73 (0.52) *	8.6 (0.80) *	9.85 (0.66)	8.97 (1.0) *

rPT: Peak resistive torque (nm); PT df: Peak torque 60°/s dorsiflexion; PT pf: Peak torque 60°/s plantarflexion. Data are reported as mean (SD). *: *p* < 0.05.
